# Prurigo nodularis: case series of 28 Brazilian patients treated with psychotropic medications^؆^

**DOI:** 10.1016/j.abd.2026.501347

**Published:** 2026-04-23

**Authors:** Paula Gerlero, Shirley Stefania Ilvay-Mendoza, Alexandre Jack Dwan, Marcello Menta Simonsen Nico

**Affiliations:** Department of Dermatology, Faculty of Medicine, Universidade de São Paulo, São Paulo, SP, Brazil

Dear Editor,

Prurigo nodularis (PN) is a chronic, intensely pruritic dermatosis of unknown cause. PN develops predominantly in adults,^1^ affecting men and women equally; in a US study, 53.1% were women, while 46.9% were men.^2^ The pathophysiology of PN remains unknown; mechanisms of neuronal sensitization and a cycle of itching and scratching contribute to the chronicity of the disease, with a clear influence of emotional factors.^1^

PN is defined as an intense, chronic pruritic condition with the presence of numerous localized or generalized papulo-nodular lesions. They are distributed symmetrically in areas of the skin accessible to scratching, being more common on the length of the limbs. A characteristic sign is the absence of lesions in areas inaccessible to scratching, such as the upper back, demonstrating that lesions are self-inflicted. Nodules present with a rough, sometimes excoriated surface and are generally dark in color. Most PN patients report a combination of sensations ranging from heat and cold to stinging, burning, and tingling.^3^

PN can occur in healthy individuals or in conjunction with systemic diseases: kidney disease, hepatitis C, obstructive pulmonary disease, congestive heart failure, and atopic diathesis (AD).^4^ It is also associated with advanced HIV infection and mental health disorders.^2^ Anxiety occurs in 37% of patients, followed by depression in 29%, and suicidal ideation in 19%.^5^ It is believed that patients with PN have a threefold increased risk of depression compared to other inflammatory dermatoses. Furthermore, the severity of depression has been shown to have a direct impact on the intensity of pruritus, highlighting the importance of intervening in the mental state.^6^

Treatment of PN is challenging. Recommendations include topical corticosteroids, capsaicin, calcineurin inhibitors, phototherapy, and systemic gabapentinoids, μ-opioid receptor antagonists, antidepressants, immunosuppressants, or biologics such as dupilumab.^7^ Our psychodermatosis group approaches the disease as being entirely secondary to scratching due to uncontrollable pruritus. We therefore present our findings on the treatment of PN with psychotropic medications that modify the sensation of pruritus.

Our objective is to describe the clinical characteristics and follow-up of 28 patients with PN seen at the Psychodermatology Clinic of the Department of Dermatology, Hospital das Clínicas, University of São Paulo, Brazil. We also present our findings on the treatment of PN with psychotropic medications that modify the sensation of pruritus.

We conducted a retrospective case series study of patients with PN from 2012 to 2024. Patients were included if they met the current diagnostic criteria: presence of firm, nodular lesions; pruritus lasting at least 6-weeks; and history or signs, or both, of repeated scratching, picking, or rubbing.^8^ Characteristics are depicted in Table 1. Our results showed a higher prevalence of women (21/28, 75%), with a mean age of 52.93 years (28‒81 years). Most patients had disseminated lesions (Figure 1, Figure 2). Skin lesions were distributed across areas accessible to hand scratching: limbs, trunk, lower back, and face. The most frequent comorbidity was hypertension (n = 7), followed by diabetes mellitus type 2 (n = 4) and previously diagnosed depression (n = 2). Lichen simplex chronicus (LSC) was the most common skin comorbidity (n = 6), followed by AD (n = 2). Skin biopsies were performed in 13 patients and revealed hyperorthokeratosis, hypergranulosis, acanthosis, dermal fibrosis, and moderate lymphohistiocytic inflammatory infiltrate in all patients. Previous treatments, which mostly included oral antihistamines combined with topical corticosteroids and emollients, were completely ineffective. The treatments instituted by our group included doxepin (doses between 10‒100 mg/day), amitriptyline (doses between 25‒100 mg/day), fluoxetine and pregabalin. The follow-up period for these patients ranged from a single visit to 14 years (mean: 887 days). Four patients were lost to follow-up before any response could be assessed (4/28). Most patients who were followed up showed a satisfactory response, ranging from partial to complete. Only a small minority did not show a favorable outcome with doxepin.

**Table 1:** Characteristics of patients with PN and their treatments.

Case	Age	Gender	Medical records	Duration of disease (m)	Associated psychiatric disease	Treatment and dosage	Time of treatment (m)	Clinical response	Follow-up (m)
1	43	F	Allergic rhinitis and asthma	95	‒	Amytriptyline 50 mg/d	73	Complete improvement	88
2	65	F	‒	64	Paranoid schizophrenia	Amytriptyline 50 mg/d	45	Complete improvement	58
3	71	M	Metabolic syndrome	36	‒	Amytriptyline 50 mg/d	9	Partial improvement	11
4	72	F	Hypertension and type II diabetes	45	‒	Amytriptyline 50 mg/d	25	Complete improvement	30
5	66	F	Hypertension	81	Bipolar disorder/ depression	Doxepin 25 mg/d	56	Complete improvement	65
6	48	F	‒	155	‒	Doxepin 25 mg/d, Fluoxetine 20 mg/d, Gabapentin 900 mg/d	96	Partial improvement	149
7	28	F	‒	67	‒	Amytriptyline 25 mg/d	38	Partial improvement	59
8	55	F	‒		‒	Doxepin 10 mg/d	‒	‒	‒
9	60	M	Epilepsy	103	‒	Doxepin 40 mg/d	87	Complete improvement	98
10	75	F	‒	20	‒	Amytriptyline 50 mg/d	3	Partial improvement	8
11	30	F	‒	49	‒	Lost follow-up	0	‒	0
12	66	M	Type II diabetes	14	‒	Lost follow-up	0	‒	0
13	40	F	Atopic dermatitis	12	‒	Lost follow-up	0	‒	0
14	64	M	Hypertension/ type II diabetes	75	‒	Amytriptyline 75 mg/d	56	Complete improvement	63
15	53	F	Chronic venous insuficiêncy	8	‒	Doxepin 20 mg/d	1	‒	1
16	30	F	‒	86	‒	Doxepin 20 mg/d	72	Partial improvement	76
17	81	F	‒	95	‒	Doxepin 15 mg/d	78	Complete improvement	88
18	61	M	Rheumatoid arthritis	14	‒	Doxepin 20 mg/d	8	Poor response	11
19	79	F	Hypertension/ dyslipidemia	84	‒	Doxepin 20 mg/d	67	Poor response	75
20	44	M	‒	63	‒	Doxepin 35 mg/d	57	Complete improvement	52
21	43	F	‒	43		Amytriptyline 75 mg/d	34	Complete improvement	37
22	74	F	Hypertension	9	Depression	Lost follow-up	0		0
23	60	F	Fibromyalgia/Osteoporosis	38	‒	Fluoxetine 20‒60 mg/d, Gabapentin 1200 mg/	25	Partial improvement	27
24	29	F	Sickle cell anemia	51	‒	Amytriptyline 50 mg/d	38	Partial improvement	45
25	43	F	Rhinitis, asthma, Atopic dermatitis	9	‒	Amytriptyline 50 mg/d	1	Complete improvement	2
26	59	F	Hypertension/ type II diabetes	11	‒	Amytriptyline 50 mg/d	1	Partial improvement	2
27	54	F	‒	86	‒	Doxepin 30 mg/d	69	Partial improvement	73
28	56	M	HIV/Hypertension	20	‒	Amitriptyline 50 mg/d, Doxepin 10 mg/d	13	Partial improvement	15

M, Male; F, Female, d, Day, m, Months.

**Figure 1:**
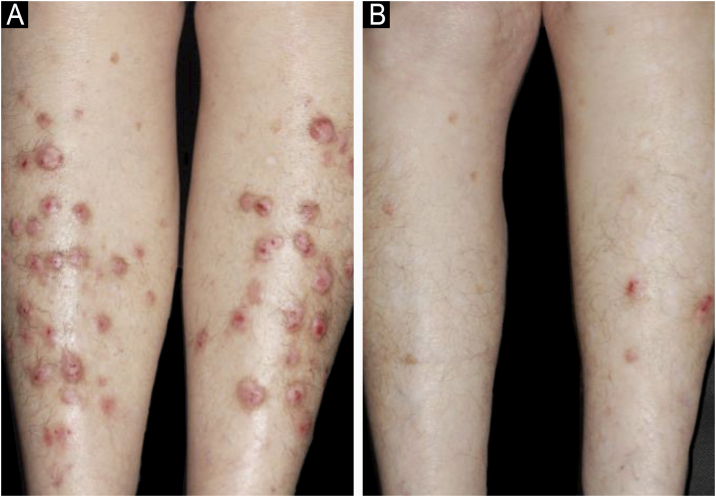
(A) Case 20 ‒ the lesions were limited to the legs in this patient. (B) Same patient after 6-months taking doxepin 35 mg/day.

**Figure 2:**
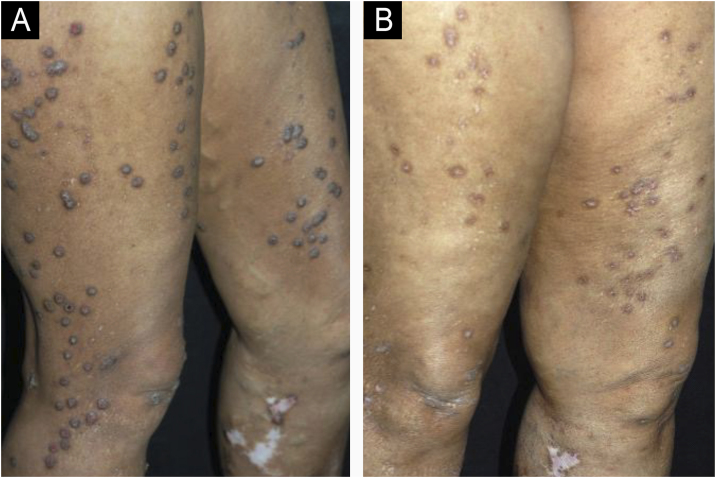
(A) Case 2 ‒ numerous PN lesions. (B) Same patient after 8-months taking amitriptyline 50 mg/day.

To our knowledge, this is the first retrospective study to evaluate clinical characteristics, long follow-up, and treatment outcomes of patients with PN in Brazil.

PN patients often present with significant psychological changes and substantially impaired quality of life. Topical treatments and antihistamines are completely ineffective for the treatment of PN, as these drugs are poor antipruritics and histamine is not implicated in the genesis of this condition.

Gabapentinoids, immunosuppressants (methotrexate, cyclosporine, azathioprine), antidepressants (mirtazapine, selective serotonin reuptake inhibitors), thalidomide, dupilumab, and other immunobiological medications are reported as being more effective. We believe that medications that reduce itching may be more effective than immunosuppressants, since lesions are secondary to scratching, and not due to a primary inflammatory phenomenon.

Tricyclic antidepressants are effective antipruritic agents due to their action on the central nervous system, even if patients do not present with obvious depression on clinical examination. Even in the latter, we observe a clear influence of mental state on pruritus, and treatment with psychotropics should be attempted. In our Psychodermatology Clinic, we have obtained excellent results with their use in various pruritic conditions (neurotic excoriations, LSC, lichen amyloidosis). Doxepin is a tricyclic antidepressant not commercially available in our setting, being purchased through compounding pharmacies. It has strong antipruritic action, with an affinity for H1 receptors 56-times greater than that of hydroxyzine and 775-times greater than that of diphenhydramine. We begin with a dose of 10 mg/day (2.5‒5 mg/day in the elderly), and we gradually increase every four weeks until the desired result is achieved. It is administered as a single nightly dose, and its main side effect is drowsiness, which can be controlled by adjusting the dose and timing of administration, so that sedation occurs in the early morning rather than during the day. Most of our cases were adequately controlled with doses less than 50 mg/day, with only a few requiring higher doses. Amitriptyline is a low-cost tricyclic antidepressant, a good alternative to doxepin, but the therapeutic range is more limited: it starts with 25 mg/day at night, and few patients tolerate doses higher than 75 mg/day due to the sedative effect.^9^ Peculiarities regarding its prescription in patients with comorbidities should be individualized on a case-by-case basis.

Serotonin reuptake inhibitors (fluoxetine, paroxetine) have less antipruritic effect than tricyclics, but can be tried in cases where there is intolerance to the latter.^10^ The mechanism of action of gabapentinoids is unclear; it is believed that they inhibit the α2δ subunit of voltage-gated calcium channels in the dorsal root ganglion and dorsal horn of the spinal cord, thus increasing the threshold for neuronal excitation by pruritic stimuli. The upper limit of the recommended dosage is 3,600 mg/day for gabapentin and 600 mg/day for pregabalin. The most common side effects are neurological symptoms such as drowsiness, dizziness, fatigue, and sedation.^11^ We only use these drugs in exceptional cases.

Adjuvant therapy for PN includes intralesional corticosteroid injection in resistant lesions. These should be injected at a high concentration (10 mg/mL of triamcinolone hexacetonide), in a small volume, and within the nodules (and not underneath), thus causing their reduction through atrophy, without compromising the surrounding skin.

Studies have shown increased expression of STAT6 in PN lesional skin, which is a marker for Th2 cells that release IL-4,10,13. These cytokines are the focus of dupilumab, which targets the IL-4 receptor, and nemolizumab, which targets the IL-31 receptor.^12^ Consistent with the central role of Th2 cytokines, blocking the IL-4/13 or IL-31 signaling pathways has demonstrated short and long-term efficacy in the treatment of PN.^5^

Limitations of our study included its retrospective design and results from a single academic center.

There are currently no therapeutic guidelines for the management of PN. As psychological factors definitively influence the course of PN (given the clear improvement in the condition with psychotropic drugs), we believe that, in the future, the association of psychotropics with modern interleukin inhibitors should be studied as a more comprehensive therapy, especially in cases where there is no response to simpler treatments.

## ORCID IDs

Shirley Stefania Ilvay-Mendoza: 0000-0002-7520-6498

Alexandre Jack Dwan: 0000-0003-3832-3190

Marcello Menta Simonsen Nico: 0000-0001-7968-0624

## Financial support

This research did not receive any specific grant from funding agencies in the public, commercial, or not-for-profit sectors.

## Authors' contributions

Paula Gerlero: Data analysis and interpretation, active participation in research supervision, intellectual participation in the therapeutic management of the case studies, final approval of the final version of the manuscript.

Shirley Stefania Ilvay-Mendoza: Study conception and design, data collection, article writing, data interpretation, critical review of the literature, and final approval of the final version of the manuscript.

Alexandre Jack Dwan: Data analysis and interpretation, active participation in research supervision, intellectual participation in the therapeutic management of the case studies, and final approval of the final version of the manuscript.

Marcello Menta Simonsen Nico: Study conception and design, data analysis and interpretation, critical review of important intellectual content, active participation in research supervision; intellectual participation in the therapeutic management of the case studies, final approval of the final version of the manuscript.

## Research data availability

The entire dataset supporting the results of this study was published in this article.

## Conflicts of interest

None declared.

## References

[1] Pereira M.P., Hoffmann V., Weisshaar E., Wallengren J., Halvorsen J.A., Garcovich S. (2020). Chronic nodular prurigo: clinical profile and burden. a european cross-sectional study. J Eur Acad Dermatol Venereol.

[2] Huang A.H., Canner J.K., Khanna R., Kang S., Kwatra S.G. (2020). Real-world prevalence of Prurigo Nodularis and burden of associated diseases. J Invest Dermatol.

[3] Pereira M.P., Steinke S., Zeidler C., Forner C., Riepe C., Augustin M. (2018). European academy of dermatology and venereology european prurigo project: expert consensus on the definition, classification and terminology of chronic prurigo. J Eur Acad Dermatol Venereol.

[4] Boozalis E., Tang O., Patel S., Semenov Y.R., Pereira M.P., Stander S. (2018). Ethnic differences and comorbidities of 909 prurigo nodularis patients. J Am Acad Dermatol.

[5] Criado P.R., Ianhez M., Criado R.F.J., Nakano J., Lorenzini D., Miot H.A. (2024). Prurigo: review of its pathogenesis, diagnosis, and treatment. An Bras Dermatol.

[6] Müller S., Zeidler C., Ständer S. (2024). Chronic prurigo including Prurigo Nodularis: new insights and treatments. Am J Clin Dermatol.

[7] Zeidler C., Pereira M.P., Ständer S. (2022). Update zur Therapie der chronischen Prurigo [Update on the treatment of chronic prurigo]. Dermatologie (Heidelb).

[8] Elmariah S., Kim B., Berger T., Chisolm S., Kwatra S.G., Mollanazar N. (2021). Practical approaches for diagnosis and management of Prurigo Nodularis: United States expert panel consensus. J Am Acad Dermatol.

[9] Zalaudek I., Petrillo G., Baldassarre M.A., De Luca T., Francione S., Sgambato A. (2006). Amitriptyline as therapeutic and not symptomatic approach in the treatment of Prurigo Nodularis. a pilot study. G Ital Dermatol Venereol.

[10] Brasileiro L.E., Barreto D.P., Nunes E.A. (2016). Psychotropics in different causes of itch: systematic review with controlled studies. An Bras Dermatol.

[11] Matsuda K.M., Sharma D., Schonfeld A.R., Kwatra S.G. (2016). Gabapentin and pregabalin for the treatment of Chronic Pruritus. J Am Acad Dermatol.

[12] Agrawal D., Sardana K., Mathachan S.R., Bhardwaj M., Ahuja A., Jain S. (2021). A prospective study examining the effect of selected topical and systemic drugs on pruritus grading system score and stat 6 expression in patients of Prurigo Nodularis. Indian J Dermatol.

